# Burnout severity in residential dementia care: individual demands and institutional-level variation in a Hungarian cross-sectional study

**DOI:** 10.1186/s12912-026-04579-y

**Published:** 2026-03-30

**Authors:** Hedvig Kiss, Zoltán Balogh, Attila Péter Szabó, Orsolya Nyilas, Péter Takács

**Affiliations:** 1 Health Sciences Division, Doctoral College, Semmelweis University, Budapest, Hungary; 2 Department of Nursing, Faculty of Health Sciences, Semmelweis University, Budapest, Hungary; 3 Department of Epidemiology, Faculty of Health Sciences, University of Debrecen, Debrecen, Hungary; 4 Institute of Applied Human Sciences, University of Nyíregyháza, Nyíregyháza, Hungary; 5 Department of Health Informatics, Faculty of Health Sciences, University of Debrecen, Debrecen, Hungary

**Keywords:** Dementia care, Burnout severity, Job demands–resources model, Coping constraints, Multilevel analysis

## Abstract

**Background:**

Burnout among staff providing residential dementia care can undermine workforce sustainability and care quality. Evidence from Hungarian long-term residential social care settings remains limited, particularly regarding the combined role of multidimensional job demands and coping-related resource constraints.

**Methods:**

A county-level cross-sectional questionnaire survey was conducted among nurses and caregivers working in residential social institutions providing dementia care in Hungary. Data collection took place between 1 June and 31 July 2024 across 77 institutions. The final sample included 1,254 participants (ordinal regression sample: 1,207). Caregiving difficulties (13 items) and dementia-specific symptom burden (12 items) were assessed on 5-point scales; internal consistency was evaluated using Cronbach’s alpha. Principal component analysis was used to develop components of caregiving difficulty. Burnout severity was operationalized as a 4-level ordinal variable outcome (0–3), derived from participants’ responses regarding their own and others’ burnout. Predictors were examined using Proportional Odds Ordinal Logistic Regression (POLR) and a multilevel Cumulative Link Mixed Model (CLMM) accounting for institutional clustering.

**Results:**

Caregiving difficulties showed excellent reliability (α = 0.928) and yielded three components explaining 73.19% of variance. Symptom burden reliability was also excellent (α = 0.949). Any burnout-related signal (levels 1–3) was reported by 46.4% of participants, while higher severity (levels 2–3) accounted for 19.3%. In adjusted ordinal models, higher subjective workload was associated with increased burnout severity (OR ≈ 1.46–1.53 across models, *p* < 0.001), as was more frequent care-related burden (OR ≈ 1.21–1.31, *p* < 0.01). Coping constraints were strongly related to higher burnout severity: reporting “no possibility to cope” was associated with increased odds (OR ≈ 2.86–2.91, *p* < 0.01). Self-reported prior learning about burnout was strongly negatively associated with burnout severity (OR ≈ 0.06–0.08, *p* < 0.001). Rest/relax frequency was not a significant predictor (OR = 1.09, 95% CI 0.95–1.24; *p* = 0.212).

**Conclusions:**

In the studied county, the level of burnout among professionals was primarily related to job expectations and limited coping options, while self-reported prior learning about burnout emerged as a potential protective factor. Organisational strategies that reduce excessive demands and expand access to coping resources—supported by targeted training—may help mitigate burnout risk in this setting.

**Supplementary Information:**

The online version contains supplementary material available at 10.1186/s12912-026-04579-y.

## Introduction

The increase in the number of dementia patients and the impact of their care on the health and social care system are highlighted in international summaries. Dementia is a growing public health burden worldwide: the WHO estimates that 57 million people were living with dementia in 2021, and nearly 10 million new cases are emerging each year [1, 2]. In long-term residential care, care needs are often complex and ongoing. The daily work of nursing and caregiving involves physical, cognitive and emotional strain. This is critical for nursing and social care, as persistent strain can affect workforce retention, continuity of care and safety of care. The role of nurses and caregivers working in residential Long-Term Care (LTC) settings is crucial, so exploring the strains on their work is essential.

According to the burnout theoretical framework, the emotional and organizational burden of care work can be associated with the development/intensification of burnout. Its three main dimensions are emotional exhaustion, cynicism/depersonalization and reduced (personal/professional) effectiveness [3]. Based on international evidence, burnout is not just a workplace well-being issue: several studies indicate a relationship between staff well-being/burnout and the safety and quality of care [4]. Studies conducted specifically in long-term care units have shown that burnout indicators of nurses and caregivers can also be related to objective quality indicators (e.g. frequency of complications/negative events) [5, 6]. This relationship is particularly relevant in the care of people with dementia, where care situations often involve a high emotional and behavioral management burden, while care situations are often unpredictable and resource-intensive.

Behavioral and Psychological Symptoms of Dementia (BPSD) – such as agitation, aggression, psychotic symptoms, sleep disturbances, wandering, repetitive questioning – are very common during the course of the disease and are one of the most important sources of burden in everyday care [7]. These symptoms not only increase care time and attention demands, but can also generate communication difficulties, safety risks and conflict situations. This can increase the psychological burden of caregivers and therefore it is justified to build on a separate measurement of dementia-specific symptom burden (rather than just “general stress”), because this can be more directly linked to care processes and targeted intervention points.

The Job Demands–Resources (JD-R) model provides a widely used framework for explaining burnout and persistent job strain. It posits that high job demands (e.g. overload, time pressure, emotional strain) combined with insufficient resources (e.g. limited organizational support, restricted opportunities for competence development, inadequate rest and recovery) jointly increase the risk of burnout [8–10]. In the present study, the JD-R framework serves as the conceptual structure for distinguishing between job demands (caregiving difficulties, dementia-related symptom burden, and workload characteristics) and job/individual resources (coping strategies, recovery opportunities, and burnout-related knowledge), with burnout severity conceptualised as the outcome variable.

In parallel, the classic framework of stress-coping suggests that the cognitive appraisal of stressful situations and the available coping resources (e.g. active strategies, social support, professional help) can influence the consequences of stress [11]. In residential institutional settings, staff support is not only an individual issue, but also a management and organizational issue. Some articles in the literature explore the organizational challenges of implementing person-centered leadership and indirectly shed light on why protective resources can be compromised in everyday operations [12, 13].

In the international literature, it is common to examine burnout alone or to highlight only one group of stressors (general strain, organizational factors or symptom strain). However, systematic summaries of the occurrence and correlations of stress and burnout among workers in long-term care facilities for people with dementia also suggest that the phenomenon can be understood as the combined result of several factors (strain, BPSD-related challenges, organizational environment, support and individual factors) [14]. Consequently, there is a special value in studies that measure (i) the multidimensional structure of caregiving difficulties, (ii) dementia-specific symptom strain, (iii) everyday workload–frequency–rest/recovery characteristics, and (iv) potentially modifiable factors such as coping and learning in the same sample.

This study examines patterns of caregiving difficulties and dementia-specific symptom burden and their relationship with burnout severity in a large sample of nurses and caregivers working in residential dementia care in Hungary (Szabolcs-Szatmár-Bereg county). By including multiple institutions of varying capacities, the study captures heterogeneity at both individual and institutional levels.

The primary objective was to investigate burnout severity (four-level ordinal outcome, 0–3) within the theoretical Job Demands–Resources (JD–R) framework and to identify its individual- and institutional-level determinants. Job demands were operationalised as multidimensional care-related strain requiring sustained physical, cognitive, or emotional effort in everyday dementia care. These included organisational and competency-related caregiving difficulties, patient-related burden associated with dementia-specific symptoms, and subjective workload characteristics. Individual resources were represented by coping-related variables and prior learning about burnout, while organisational characteristics reflected structural context.

Based on the JD–R framework, it was hypothesised that higher job demands would be associated with greater burnout severity, whereas resource-related factors would demonstrate protective effects. To test these assumptions, the study first examined the dimensional structure of caregiving difficulties and subsequently modelled the independent and combined associations of demand and resource variables with burnout severity using ordinal regression and multilevel analysis accounting for institutional clustering.

By integrating individual- and institutional-level perspectives, the study aimed to provide empirically grounded insight into modifiable risk and protective factors in residential dementia care.

## Methods

### The data collection process

The data collection was carried out in two phases. First, the measurement tool (questionnaire) was developed and verified through a paper-based pilot study conducted in two institutions (*N* = 42) to assess clarity and completeness. Based on participant feedback, only minor modifications were required. Following the pilot phase, the main data collection was conducted between June 1, 2024, and July 31, 2024. Participants were fully informed about the aims of the study and the use of the data. Participation was voluntary, informed consent was obtained from all participants, and anonymity was ensured throughout the research process. The study was conducted in accordance with the Declaration of Helsinki. The research protocol was approved by the Ethics Committee of the Ministry of the Interior, Hungary (approval number: BM/8455-3/2024).

During the preparation of the study, the heads of 97 residential social institutions in Szabolcs Szatmár Bereg County, Hungary, were contacted. Of the 97 institutions, 78 returned a statement of intent. In the end, the survey was carried out in 77 institutions, as there was a change of leadership in one institution during the research and the new leader no longer wished to participate in the research. A contact person was designated in the institutions participating in the research to conduct the data collection.

At the start of the survey, the online link to the questionnaire was sent directly to the institutional contact person. The contact persons organized the completion among the staff in the institutions. The completion of the questionnaire was voluntary, and the data were recorded anonymously at the central address. Participants were nurses and caregivers working in residential social institutions providing care for people living with dementia. Before completing the questionnaire, the participants received written information and a consent form template. After their consent and completion, one copy of the consent form remained with the participant, and one copy was sent to a designated member of the research team. The consent form did not contain a questionnaire identifier and could not be linked to the responses.

The sampling cannot be considered random due to the process detailed above. The survey attempted to reach all employees of the entire county healthcare system. A total of *n* = 1,428 people responded to the questionnaire. The nominal number of professional staff in the 77 institutions surveyed was 1,454 - the response rate: 98.21%.

The questionnaire asked about the respondent’s direct care activities. 122 people did not answer this question (the status of direct care participation could not be determined), and 52 people indicated that they were not directly involved in care - they were excluded from further analyses. The study sample was then fixed at 1,254 people after the exclusion step.

### Measures and variables

The questionnaire was compiled after a literature review. The topics of previous dementia care surveys were adapted to create our own questionnaire – adjusted to local (national) characteristics, such as educational level, institutional size, etc.

The first part of the self-designed, 30-item questionnaire included sociodemographic questions (gender, age, highest professional qualification, dementia-specific qualifications, professional experience, location of the institution and number of beds). This was followed by statements aligned with the researcher’s hypotheses, mainly rated on a 1–5 Likert scale, or binary choice questions to examine knowledge, attitudes, care conditions and personal characteristics related to dementia. Several questions also had the option of open-ended responses; brief instructions were provided to assist in completing them. In more detail (Qx indicates the question numbering in the questionnaire):

The questionnaire was developed to reflect the practical realities of residential dementia care in the Hungarian long-term social care context. Existing standardized instruments were reviewed; however, many did not fully capture dementia-specific care challenges and institutional conditions relevant to the target population. Therefore, context-adapted items were formulated to ensure practical comprehensibility for frontline caregivers and to allow direct linkage between care processes and burnout-related outcomes. The instrument underwent pilot testing in two institutions (*N* = 42), and internal consistency indices for multi-item domains were subsequently evaluated in the main sample.

- **Sociodemographic and work-context variables.** Gender was recorded as female/male. Age group was recorded in five categories (21–30, 31–40, 41–50, 51–60, 61–70 years). Highest education was collected as a multiple-response item and used to describe the sample (multiple qualifications could be marked). Years of experience in social care as a nurse/caregiver were recorded in ordered categories (< 1; 1–5; 6–10; 11–20; 21–30; 31–40; > 40 years). During analysis, the 31–40 and > 40 experience groups were merged into a single “31 + years” category. Facility location was recorded as city / commune / village; for analyses it was recoded into city vs. rural = commune or village. Facility capacity was recorded in four categories (1–25, 26–50, 51–100, ≥ 100 beds/places).
- **Caregiving difficulties (13 items).** Caregiving difficulties were assessed using a 13-item scale rated on a 5-point Likert scale (1 = lowest difficulty; 5 = highest difficulty). The item set was used to derive a multidimensional structure (component scores, F1, F2, F3) for subsequent analyses. Conceptually, these items were designed to capture multidimensional care-related demands operating at organisational, task-related, and individual strain levels, consistent with the demand component of the JD–R framework.
- **Dementia-related symptom burden (12 items).** Perceived burden of dementia-related symptoms was measured with 12 items rated on a 5-point Likert scale (1 = lowest burden; 5 = highest burden). The symptom items were summarised into a single regression-based component score (F4) for group comparisons and further analyses.
- **Workload**,** difficulty frequency**,** and recovery opportunity.** Perceived workload was measured with three 5-point items (physical, mental, psychological; 1 = lowest, 5 = highest). A composite mean workload score was used in the model. The three workload items (physical, mental, psychological strain) showed strong positive intercorrelations (see Results). Therefore, a composite workload index was calculated as the arithmetic mean of the three items, with higher values indicating higher overall perceived workload. Frequency of experiencing difficulties was recorded as an ordered categorical variable (rarely; weekly 1–2 times; every day; does not experience difficulties) and coded so that higher values reflected higher frequency. Opportunity to rest after work was recorded in four ordered categories (daily; weekly 1–2 times; a few times per month; no opportunity). For analyses, higher values indicated less frequent rest (relax).
- **Coping strategies and derived coping typology**. Coping strategies were assessed with a multiple-response item listing several behavioural and support-seeking options. For modelling, responses were recoded into a 3-category coping typology: Active coping (endorsed at least one adaptive behavioural or support-seeking strategy such as sport, hobby, setting work–family boundaries, social support, community activity, or professional support), No possibility (“no opportunity; too busy”), No need (“does not think such activities are needed”).
  Importantly, the “No possibility” category was interpreted as an indicator of restricted access to coping resources rather than absence of coping skills. Accordingly, within the JD–R framework, this variable was treated as a perceived resource-access constraint.

- **Burnout-related indicators and derived burnout severity.** Burnout-related knowledge and self/other-observed signs were captured by a multiple-response item. A binary indicator “burnout-related learning” was created from the option “Yes, I have learned/read about it” (coded 1 = yes, 0 = no). The primary outcome, burnout severity, was operationalised as a 4-level ordinal variable (0–3): 0 = no signal, 1 = mild signal (observed in colleagues), 2 = self-noticed signs, 3 = self-noticed signs + thoughts about career change/exit. The composite score was constructed according to the highest endorsed category across the burnout-related response options.

### Statistical analysis

In the data file, records were separated by a unique identifier; duplicates and obvious technical fillings were deleted. Data processing was performed with IBM SPSS Statistics 23.0 and R 4.1.1. (packages used: RcmdrMisc; MASS; dplyr, cat; brant; ggplot2; broom; VGAM, clubSandwich, ordinal, openxlsx).

Descriptive statistics were used to summarise participant characteristics and study variables (frequencies and percentages for categorical variables; means and standard deviations, with 95% confidence intervals where applicable). Group differences between categorical variables were examined using Pearson’s chi-square test (or Fisher’s exact test when expected cell counts were small), and effect size was quantified using Cramer’s V.

Internal consistency of multi-item scales was evaluated with Cronbach’s alpha. The suitability of the correlation matrices for dimension reduction was assessed using the Kaiser–Meyer–Olkin (KMO) measure and Bartlett’s test of sphericity. Principal component analysis (PCA) with Varimax rotation (Kaiser normalisation) was applied to the 13-item caregiving difficulties set, and regression-based component (factor) scores were computed for subsequent analyses.

Between-group comparisons of component/factor scores (and other derived scores) across institutional capacity categories and experience groups were conducted using one-way ANOVA. Homogeneity of variances was checked using Levene’s test; when violated, Welch’s robust ANOVA and Tamhane post-hoc tests were used. As a non-parametric sensitivity analysis, Kruskal–Wallis tests were performed; where relevant, Monte Carlo p-values were reported to confirm robustness.

Because several variables were ordinal and/or showed non-normal distributions, associations between perceived physical, mental, and psychological workload, frequency of difficulties, and opportunities for rest were primarily evaluated using Spearman’s rank correlation (ρ).

Predictors of burnout severity (ordinal outcome, 0–3) were examined using Proportional Odds Ordinal Logistic Regression (POLR). The model included regression-based component scores derived from factor analysis (F1, F2, F4), capacity, perceived workload, frequency of difficulties (frequency), opportunity for rest (relax; higher values indicate less frequent rest), burnout-related learning (learn), age group, gender, years of experience (categorical), settlement type, and coping category (reference: active coping). Cases with missing data on model variables were excluded listwise (final analysed *N* = 1,207). Model results were reported as odds ratios (OR = exp[b]) with 95% confidence intervals based on profile likelihood, and overall fit was described using residual deviance and AIC. Multicollinearity was assessed using GVIF^(1/(2·Df)) - values below 2 were considered indicative of no relevant multicollinearity. The proportional odds assumption was evaluated using the Brant test.

**Fig. 1:**
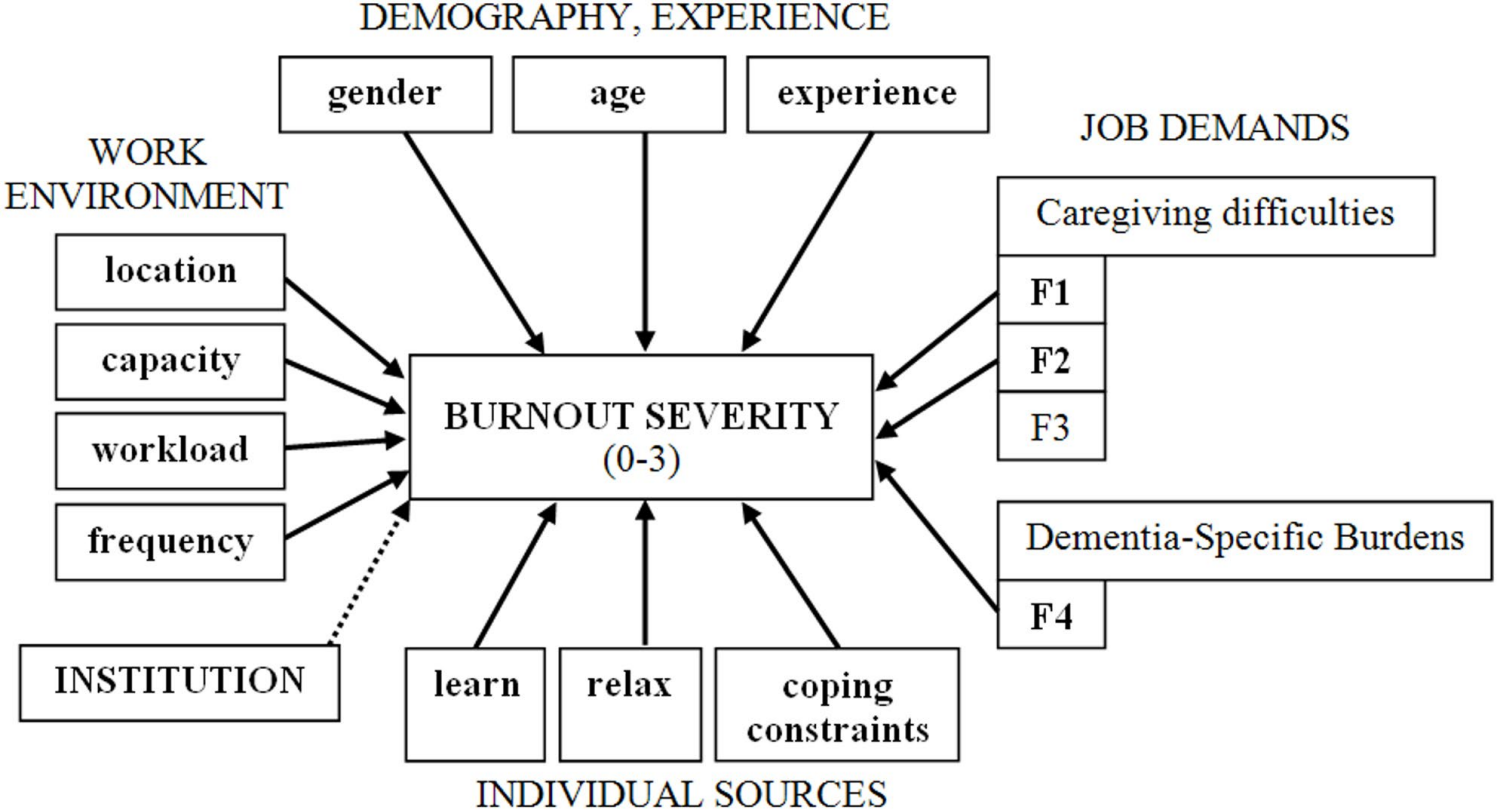
Cumulative Link Mixed Model of examined Burnout Severity. Notes: The Fig. 1 illustrates the multilevel conceptual framework of the study. Individual-level job demands included caregiving difficulty components (F1, F2), dementia-specific symptom burden (F4), perceived workload (composite index), and frequency of difficulties. Individual-level resources included coping typology, burnout-related learning, and opportunity for relaxation. Burnout severity (0–3 ordinal outcome) was specified as the dependent variable. Institutional clustering was modelled using a random intercept to account for between-facility variance

Because respondents were nested within institutions, a multilevel Cumulative Link Mixed Model (CLMM) with a random intercept for institution was fitted as a secondary model. Institutional clustering was specified as a random effect to account for non-independence of observations within facilities. The conceptual structure of the multilevel model and the relationships between examined domains and burnout severity are illustrated in Fig. 1 (see Results section G.2).

The variance of the random intercept was used to compute the Intraclass Correlation Coefficient (ICC) following the latent variable approach for ordinal models. Model fit between the single-level proportional odds model and the multilevel CLMM was compared using AIC (Akaike information criterion).

Although data collection was conducted in 77 institutions, several institutions operated multiple member facilities. Therefore, the clustering variable (institution_id) reflected the organisational unit at the facility level, resulting in a higher number of level-2 units in the multilevel model. In total, responses were clustered within 87 organisational units in the multilevel analysis.

All tests were two-sided and statistical significance was set at *p* < 0.05.

## Results

### Demographic and professional characteristics of the sample

The sample size was *N* = 1,254. The majority of respondents were female (93.3%, *n* = 1,170), while 6.7% were male (*n* = 84).

The majority of participants were middle-aged: 36.8% were between 41 and 50 years old, and 34.3% were between 51 and 60 years old. Together, these two groups accounted for 71.1% of the sample. The youngest group (21–30 years old) accounted for 5.8%, and those over 60 accounted for 6.9%. There was a small but statistically significant correlation between gender and age group (χ²(4, *N* = 1,254) = 18.52, *p* = 0.001; Fisher’s exact test *p* = 0.003, Cramer V = 0.12, *p* = 0.001). This indicated smaller distributional differences between age groups.

In terms of professional experience, 75.4% of respondents had worked in social care for at least 6 years, and 54.6% had more than 10 years of experience. The largest subgroup was those with 11–20 years of experience (28.5%). There was no significant correlation between gender and years of professional experience (χ²(5, *N* = 1,254) = 3.69, *p* = 0.595).

In the case of the question on professional education, respondents could indicate more than one qualification. The two most common qualifications were social nurse and caregiver (based on primary education) and social nurse and caregiver (based on high school diploma). Together, these accounted for 76.2% of the sample. Higher education (BSc and MSc combined) represented 11.3%.

Considering the institutional characteristics, 51.5% of the participants worked in urban institutions (*n* = 646) and 48.5% in rural institutions (*n* = 608).

Regarding institutional capacity, 42.5% worked in institutions with 26–50 beds, 20.2% in institutions with 51–100 beds, 24.3% in institutions with more than 100 beds, and 13.0% in institutions with 1–25 beds. A significant but small correlation was observed between settlement type and institutional capacity (χ²(3, *N* = 1,254) = 14.60, *p* = 0.002; Cramer V = 0.108, *p* = 0.002).

All of these descriptive data are summarized in more detail in Table 1.

**Table 1:** Demographic and professional characteristics of the sample (*N* = 1,254)

Variable	Category	*n*	%
Gender	Male	84	6.7
	Female	1,170	93.3
Age group (years)	21–30	73	5.8
	31–40	203	16.2
	41–50	462	36.8
	51–60	430	34.3
	61–70	86	6.9
Professional experience	< 1 year	57	4.5
	1–5 years	251	20.0
	6–10 years	261	20.8
	11–20 years	358	28.5
	21–30 years	258	20.6
	31 + years	69	5.5
Qualification*	Social care and nursing (primary education)	484	—
	Social worker and nurse (high school)	472	—
	Health vocational school	68	—
	Health vocational school with high school diploma	148	—
	College (BSc)	114	—
	University (MSc)	27	—
	Dementia care professional qualification	324	—
	Other health or social care qualification(s)	151	—
Settlement type	Urban	646	51.5
	Rural	608	48.5
Institution size/capacity (beds)	1–25	163	13.0
	26–50	533	42.5
	51–100	253	20.2
	100+	305	24.3

* Multiple response variable; percentages do not sum to 100%

### The structure of caregiving difficulties

#### Psychometric properties and dimensional structure

Respondents rated 13 caregiving difficulties on a scale of 1–5 (1 = lowest, 5 = highest) in the questions. The mean of the items ranged from 2.41 to 3.60 (SD = 1.24–1.35), indicating an overall moderate level of difficulty. The highest mean was reported for constant readiness and attention (M = 3.60), followed by physical strain related to caring for bedridden patients (M = 3.55) and sleep-wake disorders that interfered with work schedules (M = 3.42). The lowest means were reported for mental burnout (M = 2.41) and physical exhaustion (M = 2.53).

Due to the relatively large number of items in the caregiving difficulties section of the questionnaire, Principal Component Analysis was used to create a smaller number of conceptually coherent dimensions (Table 2).

The internal consistency of the 13-item scale into the complex model was excellent (Cronbach-α = 0.928; *N* = 1,251, 99.8% valid cases). Sampling adequacy was confirmed (KMO = 0.906), and the Bartlett test was significant, χ²(78) = 11776.91, *p* < 0.001. Principal Component Analysis with Varimax rotation resulted in a three-component solution. This model explained 73.19% of the total variance (Component 1: 31.13%; Component 2: 25.91%; Component 3: 16.16%).

The F1 component reflected the organizational/resource and competency conditions. Component F2 captured patient-related care burdens and challenges in organizing care. Component F3 measured subjective fatigue/burnout (this component was not included in the complex models to avoid conceptual overlap with the outcome variable).

**Table 2:** Rotated component matrix of caregiving difficulties

Rotated Component Matrix^a^			
	Component		
	1	2	3
Insufficient material conditions for special care.	**0.826**	0.321	0.097
Inadequate environmental conditions for special care.	**0.814**	0.315	0.131
The practical professional knowledge of the care providers is insufficient.	**0.763**	0.255	0.217
There is no proper cooperation between colleagues.	**0.710**	0.176	0.320
Inadequate personnel conditions, low number of care providers.	**0.645**	0.489	0.018
Lack of modern theoretical knowledge about the disease and its specific care.	**0.628**	0.203	0.312
Insufficient employment and daily schedule for patients.	**0.562**	0.479	0.220
The special care needs of patients with difficult-to-control, disturbed consciousness require constant readiness and attention.	0.284	**0.858**	0.132
Sleep-wake disorder, which often occurs in patients, disrupts work schedules.	0.209	**0.835**	0.209
They represent a serious physical strain due to caring for bedridden patients who are unable to care for themselves.	0.306	**0.820**	0.104
Lack of cooperation from relatives or their rejection due to lack of knowledge.	0.408	**0.624**	0.181
I was mentally tired of this job, I was burned out.	0.221	0.149	**0.923**
I’m physically tired of this work, I can’t take this strain anymore.	0.238	0.204	**0.906**

Extraction Method: Principal Component Analysis. Rotation Method: Varimax with Kaiser Normalization

a. Rotation converged in 6 iterations

##### Institution size and caregiving difficulty factors

Significant differences were found between institutional capacity categories for F1 (Welch F(3, 522.58) = 7.34, *p* < 0.001; η² = 0.016) and F3 (Welch F(3, 516.59) = 11.22, *p* < 0.001; η² = 0.025), while F2 was not significant (F(3, 1247) = 1.75, *p* = 0.154; η² = 0.004).

For F1, respondents working in institutions with 1–25 beds had lower scores than those working in institutions with 26–50 beds (mean difference = − 0.395, *p* < 0.001).

For F3, respondents working in institutions with more than 100 beds had significantly lower scores than all smaller capacity categories (1–25 beds: difference = 0.336, *p* = 0.005; 26–50 beds: difference = 0.389, *p* < 0.001; 51–100 beds: difference = 0.301, *p* = 0.001, 95%).

Nonparametric Kruskal–Wallis analyses confirmed these patterns: significant differences were observed for F1 (H(3) = 18.76, *p* < 0.001) and F3 (H(3) = 29.23, *p* < 0.001), but not for F2 (H(3) = 5.77, *p* = 0.123).

##### Work experience and caregiving difficulty factors

No significant differences were found across work experience groups for F1 (F(5, 1245) = 0.95, *p* = 0.449; η² = 0.004) or F2 (F(5, 1245) = 2.00, *p* = 0.075; η² = 0.008). In contrast, F3 differed significantly by work experience (F(5, 1245) = 4.04, *p* = 0.001; η² = 0.016). Respondents with 1–5 years of experience showed lower F3 scores compared to those with 6–10 years (MD = − 0.283, *p* = 0.012), 11–20 years (MD = − 0.298, *p* = 0.003), and 21–30 years (MD = − 0.340, *p* = 0.002).

Kruskal–Wallis tests yielded consistent results: no significant differences for F1 (H(5) = 5.19, *p* = 0.394) or F2 (H(5) = 10.14, *p* = 0.071), but significant differences for F3 (H(5) = 20.23, *p* = 0.001).

### Dementia-specific symptom burden

#### Internal consistency and scale characteristics

Respondents rated 12 dementia-related symptoms on a 1–5 scale. Item means ranged between 3.13 and 3.65 (SD = 1.17–1.45), indicating moderate to high perceived burden. The highest mean was observed for confusion (M = 3.65), followed by physical deterioration/immobility (M = 3.62) and wandering (M = 3.60). The lowest mean was reported for neglect of personal hygiene (M = 3.13).

Internal consistency was excellent (Cronbach’s α = 0.949; *N* = 1,251, 99.8% valid cases). Sampling adequacy was high (KMO = 0.939), and Bartlett’s test was significant, χ²(66) = 13640.76, *p* < 0.001.

No significant differences in symptom burden were found by settlement type (F(1, 1249) = 0.30, *p* = 0.582; H(1) = 0.46, *p* = 0.499, η² = 0.0002) or by work experience (F(5, 1245) = 2.15, *p* = 0.057, η² = 0.0002).

### Workload, frequency of difficulties and opportunity to relax

Respondents rated physical, mental, and psychological strain on a scale of 1–5 (*N* = 1,252 valid cases). The mean scores were as follows: physical strain M = 3.39 (SD = 1.24), mental strain M = 3.46 (SD = 1.20), and psychological strain M = 3.33 (SD = 1.26). Mental strain showed the highest central tendency. The distributions were moderately skewed toward higher categories.

Spearman correlations (all *p* < 0.001) indicated strong positive associations between the three workload dimensions (physical–mental ρ = 0.717; physical–psychological ρ = 0.647; mental–psychological ρ = 0.868). Given these substantial intercorrelations and the conceptual coherence of the items, the three dimensions were combined into a composite workload index calculated as their arithmetic mean. This composite score was subsequently used in the regression models.

Regarding the frequency of difficulties, 92.2% of respondents reported at least occasional difficulties, and 26.8% reported daily difficulties.

Less favorable opportunities to relax were reported by 28.3% of participants, while 71.7% indicated at least weekly opportunities for rest.

Frequency of difficulties showed a moderate positive correlation with workload (ρ = 0.359–0.429), and fewer opportunities to relax were also associated with higher workload (ρ = 0.302–0.356). A small but statistically significant association was observed between frequent difficulties and reduced relaxation opportunities (ρ = 0.278, *p* < 0.001).

### Coping strategies

A total of 1,213 respondents (96.7%) provided valid responses regarding coping strategies. The most frequently mentioned coping strategy was prioritizing family life and avoiding taking work home (*n* = 772; 63.6%). This was followed by hobbies (*n* = 448; 36.9%) and sports/physical recreation (*n* = 394; 32.5%). Friends and social programs were mentioned by 26.8% (*n* = 325). Professional help was rare (*n* = 42; 3.5%), as was community participation (*n* = 29; 2.4%).

Based on the composite typological variable (*N* = 1,207 valid cases), 93.5% of respondents fell into the active coping category (*n* = 1,128). The “I have no way” category was indicated by 3.7% (*n* = 45). 2.8% (*n* = 34) reported that they did not need coping activities. Overall, 6.5% fell into the less favorable coping categories.

### Burnout severity

A total of 1213 respondents (96.7%) provided valid answers to the burnout severity question group. The majority of respondents reported their existing knowledge of burnout symptoms (*n* = 876; 72.2%). This question was later included as a predictor variable in the complex model.

Signs of burnout were perceived by 36.7% (*n* = 445) of their colleagues and 18.3% (*n* = 222) of themselves. Thoughts of career change were rare (*n* = 27; 2.2%). The severity of burnout was described with a four-level ordinal composite variable (0–3), which took into account the highest marked value. The distribution was as follows:

- 0 (no symptoms): 650 respondents (53.6%).
- 1 (mild symptoms in others): 328 (27.0%).
- 2 (self-perceived symptoms): 208 (17.1%).
- 3 (self-perceived symptoms with thoughts of career change): 27 (2.2%).

Overall, 46.4% of respondents reported some level of burnout-related symptoms (categories 1–3), and 19.3% fell into the more severe categories (2–3).

### Multivariate modeling of burnout severity

#### Proportional Odds Logistic Regression (POLR) model

Burnout severity was analyzed as a four-level ordinal outcome variable in the first stage of the analysis using proportional odds logistic regression (POLR). The model included factors of caregiving difficulties (F1, F2), dementia symptom burden (F4), coping categories, subjective workload, frequency of difficulties, opportunity for relax, burnout-related learning, and relevant sociodemographic and institutional characteristics (gender, age, professional experience, settlement type, institution capacity).

The results of the single-level model showed that burnout-related learning significantly and strongly reduced the odds of belonging to the higher burnout category. In contrast, the category “I have no way, I am very busy” (no way) significantly increased the odds of burnout severity. Higher subjective workload and more frequent occurrence of caregiving difficulties also showed a positive association with burnout severity.

The first and second factors of caregiving difficulties (F1, F2) and the composite factor of dementia symptom burden (F4) did not prove to be significant predictors in the full model. In terms of years of experience, the middle-career groups (experience 11–20 years; experience 21–30 years) showed an increased chance of higher burnout categories.

In addition, respondents who answered “I do not think I need any such activities” (not necessary) in the coping question; and those who worked in a care facility with a larger capacity (capacity > 100 people; capacity 51–100 people) were classified in a significantly lower risk group (Table 3).

**Table 3:** Results of proportional odds logistic regression model

	Value	Std. Error	t value	*p* value	OR	CI_low	CI_high
learn ↓	-2.6020	0.1484	-17.5351	0.0000	0.0741	0.0552	0.0988
F1	0.1284	0.0681	1.8846	0.0595	1.1370	0.9949	1.2997
F2	-0.0242	0.0772	-0.3131	0.7542	0.9761	0.8393	1.1360
F4	0.0690	0.0848	0.8144	0.4154	1.0715	0.9069	1.2646
coping no way ↑	1.0692	0.3106	3.4426	0.0006	2.9132	1.5769	5.3434
coping not necessary ↓	-0.8351	0.4184	-1.9957	0.0460	0.4339	0.1843	0.9601
workload ↑	0.3784	0.0766	4.9382	0.0000	1.4600	1.2575	1.6984
frequency ↑	0.1944	0.0761	2.5565	0.0106	1.2146	1.0467	1.4105
relax	0.0767	0.0682	1.1255	0.2604	1.0797	0.9443	1.2337
gender female	-0.4062	0.2537	-1.6012	0.1093	0.6662	0.4070	1.1021
age 31–40	0.1235	0.3258	0.3792	0.7046	1.1315	0.6034	2.1710
age 41–50	0.0150	0.3157	0.0477	0.9620	1.0152	0.5528	1.9119
age 51–60	0.3355	0.3245	1.0339	0.3012	1.3987	0.7483	2.6783
age 61–70	0.2474	0.3914	0.6321	0.5273	1.2806	0.5976	2.7786
experience 1–5 years	-0.0151	0.3310	-0.0457	0.9636	0.9850	0.5201	1.9105
experience 6–10 years	0.5755	0.3303	1.7427	0.0814	1.7781	0.9417	3.4489
experience 11–20 years ↑	0.8370	0.3318	2.5225	0.0117	2.3094	1.2204	4.4967
experience 21–30 years ↑	0.8066	0.3458	2.3324	0.0197	2.2403	1.1504	4.4770
experience 31 + years	0.7496	0.4253	1.7624	0.0780	2.1161	0.9229	4.9045
location rural	-0.1099	0.1262	-0.8704	0.3841	0.8960	0.6994	1.1472
capacity 26–50 people	0.2385	0.1976	1.2071	0.2274	1.2694	0.8640	1.8761
capacity 51–100 people ↓	-0.5225	0.2240	-2.3330	0.0196	0.5930	0.3824	0.9207
capacity > 100 people ↓	-0.8253	0.2278	-3.6233	0.0003	0.4381	0.2802	0.6848

Notes: Reference categories: coping reference = “active coping”, gender reference = “male”, location reference = “city”, age reference = “21–30”, experience reference = “<1 year”, capacity reference = “1–25 people”

No multicollinearity concerns were identified (all GVIF^(1/(2*Df)) < 1.5). The proportional odds assumption was evaluated using the Brant test. The omnibus test indicated a statistically significant deviation from the parallel regression assumption (χ² = 188.13, df = 46, *p* < 0.001). Given the large sample size and the presence of sparse category combinations, this result may partly reflect the sensitivity of the test to minor departures from parallelism. Importantly, the direction and magnitude of the main effects remained stable across the single-level and mixed-effects models (see later), supporting the substantive interpretability of the estimated associations despite the formal test result.

##### Examining interactions in the model

To examine the moderating effect between the factors job demands (F2, F4) and coping constraints (coping), interaction components were included in the model (F4 × coping; F2 × coping). Based on the likelihood ratio test and AIC comparison, the interaction model did not show a significantly better fit compared to the main effects model (LR *p* = 0.1537 > 0.05; ΔAIC = 1.318 < 2). This suggests that the effect of coping constraints is more likely to be an independent main effect and does not significantly modify the effect of caregiving difficulties or symptom burden.

#### Multilevel cumulative link mixed model (CLMM)

##### Justification of institutional clustering

Since respondents came from 87 different institutions (taking into account the member institution structure), it could be assumed that individuals’ responses were not independent of each other at the institutional level. To examine this, the analysis used a Cumulative Link Mixed Model (CLMM) with an institutional random intercept. This model allows the researcher to decompose the variance in burnout severity into individual and institutional components (Fig. 1).

##### Random intercept and institutional variance

In the multilevel CLMM model, the variance of the institutional random effect was 0.854 (SD = 0.924). The intraclass correlation coefficient (ICC = 0.206) indicated that approximately 21% of the variance in burnout severity was attributable to between-institution differences. This suggests that burnout is not solely an individual-level phenomenon, but is significantly related to the institutional context.

##### Stability of fixed effects in the multilevel model

**Fig. 2:**
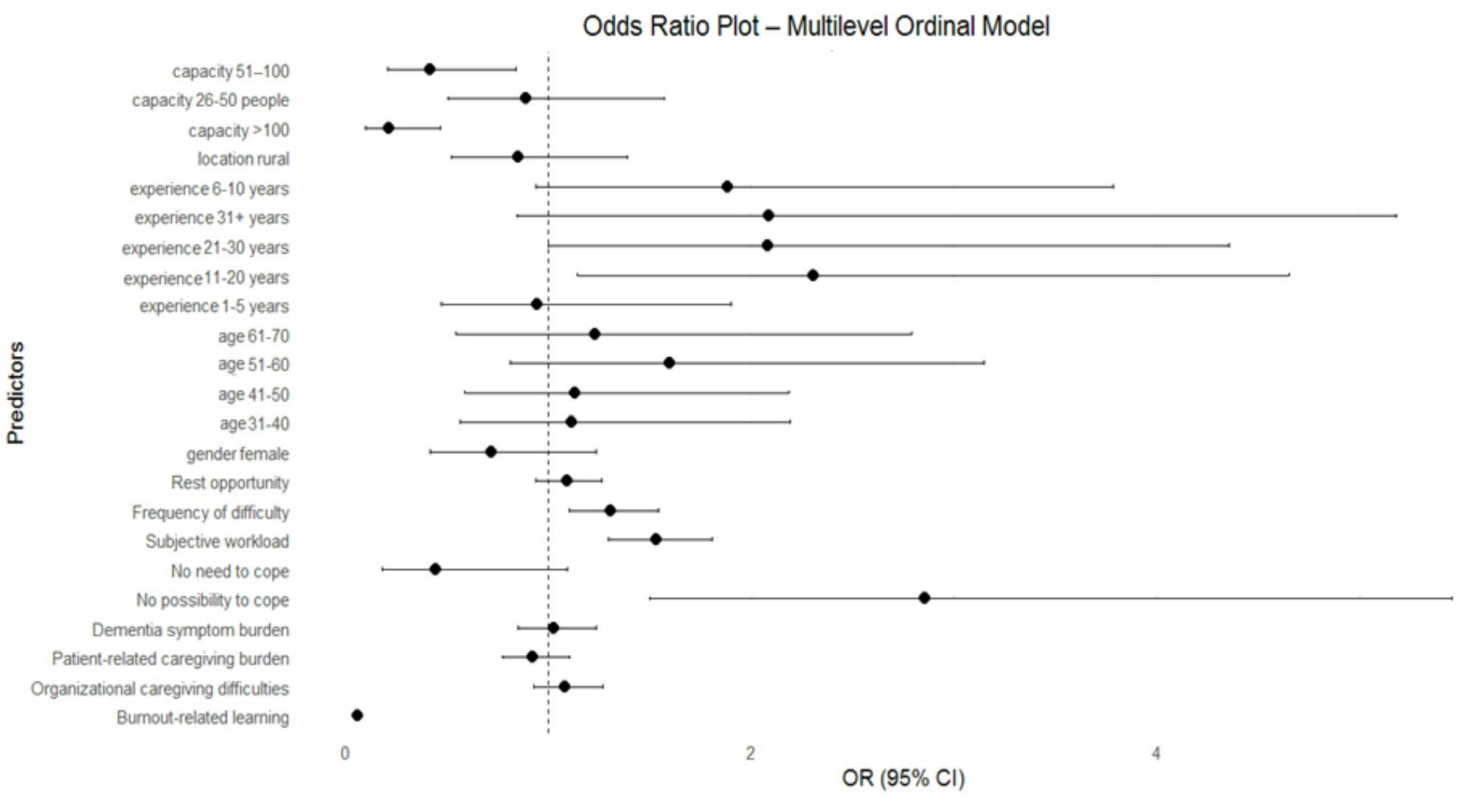
Odds ratios (OR) for Burnout Severity (0–3) CLLM model

In the multilevel CLMM model, the effects of the most important individual-level predictors remained stable in direction and magnitude. Learning about burnout continued to strongly reduce the chance of belonging to the higher burnout category. The “I have no way …” category remained a significant risk factor. Higher subjective workload and more frequent occurrence of difficulties were also significantly positively associated with the severity of burnout. However, the effects of some organizational variables changed slightly, which may indicate that they partly overlap with the institutional-level variance (Table 4; Fig. 2).

**Table 4:** Results of cumulative link mixed model

term	Estimate	Std_Error	z_value	p_value	OR	CI_low	CI_high
learn ↓	-2.8229	0.1678	-16.8241	0.0000	0.0594	0.0428	0.0826
F1	0.0810	0.0802	1.0089	0.3130	1.0843	0.9265	1.2690
F2	-0.0790	0.0897	-0.8807	0.3785	0.9240	0.7750	1.1017
F4	0.0251	0.0958	0.2619	0.7934	1.0254	0.8499	1.2371
coping no way ↑	1.0504	0.3300	3.1834	0.0015	2.8589	1.4974	5.4586
coping not necessary	-0.8069	0.4569	-1.7661	0.0774	0.4462	0.1822	1.0926
workload ↑	0.4258	0.0848	5.0216	0.0000	1.5308	1.2964	1.8075
frequency ↑	0.2664	0.0852	3.1260	0.0018	1.3053	1.1045	1.5426
relax	0.0870	0.0755	1.1518	0.2494	1.0909	0.9408	1.2650
gender female	-0.3301	0.2762	-1.1950	0.2321	0.7188	0.4183	1.2353
age 31–40	0.1078	0.3451	0.3125	0.7547	1.1139	0.5664	2.1907
age 41–50	0.1250	0.3352	0.3728	0.7093	1.1331	0.5875	2.1856
age 51–60	0.4700	0.3460	1.3585	0.1743	1.5999	0.8121	3.1520
age 61–70	0.2092	0.4171	0.5016	0.6159	1.2327	0.5443	2.7921
experience 1–5 years	-0.0569	0.3573	-0.1593	0.8734	0.9447	0.4690	1.9028
experience 6–10 years	0.6332	0.3562	1.7776	0.0755	1.8836	0.9371	3.7860
experience 11–20 years ↑	0.8355	0.3584	2.3315	0.0197	2.3061	1.1424	4.6551
experience 21–30 years ↑	0.7345	0.3763	1.9518	0.0510	2.0845	0.9969	4.3586
experience 31 + years	0.7368	0.4634	1.5902	0.1118	2.0893	0.8425	5.1811
location rural	-0.1587	0.2491	-0.6371	0.5241	0.8532	0.5236	1.3904
capacity 26–50 people	-0.1153	0.2887	-0.3994	0.6896	0.8911	0.5061	1.5691
capacity 51–100 people ↓	-0.8731	0.3571	-2.4447	0.0145	0.4177	0.2074	0.8411
capacity > 100 people ↓	-1.5356	0.3932	-3.9058	0.0001	0.2153	0.0996	0.4653

Notes: Reference categories: coping reference = “active coping”, gender reference = “male”, location reference = “city”, age, reference = “21–30”, experience reference = “<1 year”, capacity reference = “1–25 people”

##### Fit test of the multilevel model

The fit of the multilevel model (CLMM) improved significantly compared to the single-level model (POLR) (AIC ≈ 1994 vs. ≈ 2042, ΔAIC = 48.165). This difference suggests that including the institutional level in the model describes the data structure significantly more accurately.

#### Summary of POLR and CLMM burnout severity models

The results suggest that burnout severity is a multifactorial phenomenon, with individual-level factors – particularly subjective workload, lack of coping options and knowledge about burnout – playing a decisive role. However, institutional-level differences contribute significantly to explaining the variance, supporting the importance of the organizational context.

## Discussion

The present cross-sectional study was conducted among nurses/caregivers caring for people with dementia in residential social institutions (*N* = 1254; regression sample analyzed *N* = 1207) at the county level (Hungary, Szabolcs-Szatmár-Bereg). The analysis showed that the severity of burnout (0–3 ordinal outcome) was closely related to subjective workload (OR ≈ 1.46–1.53 across models) and to more frequent experience of difficulties (OR ≈ 1.21–1.31). These relationships remained stable in both single-level and multi-level models, supporting the robustness of the effects related to demands. In contrast, the variable indicating the rarity of relaxation did not prove to be a significant predictor in the model. Self-reported prior learning about burnout showed a strong negative association with burnout severity (OR ≈ 0.06–0.08 across models).

Consistent with the JD–R framework, caregiving difficulties and dementia-specific symptom burden were treated as multidimensional demand indicators reflecting structural, task-related, and resident-related strain. The findings therefore support the demand–exhaustion pathway described in JD–R theory.

### Care difficulties: the three-dimensional structure

The 13-item question block of caregiving difficulties was organized into three clearly distinguishable dimensions during factor analysis (organizational/resource and competence conditions; patient-side caregiving burden and care organization challenges; subjective fatigue/burnout). This result indicates that “caregiving difficulty” is not a single homogeneous phenomenon, but rather an interwoven system of burdens at multiple levels (organizational–task–personal). The three-component structure fits well with the stress–burnout patterns described in long-term, institutional dementia care. The separation of the third component (subjective fatigue/burnout) in the studies suggests that in addition to the “work environment” and “illness burdens”, an autonomous, personal exhaustion axis is also present which may represent a distinct subjective exhaustion dimension that warrants further investigation in longitudinal designs. Evidence suggests that the challenging behavior of those in care is seldom an isolated stressor; rather, staff stress reflects an interplay of resident-related factors, staff-related factors (including appraisal), and organisational context (e.g., care-home environment, perceived support and leadership). Recent literature syntheses confirm that stress/burnout is common among caregivers of people with dementia, and that multiple factors (work organization, resources, competencies, and the burden of managing challenging behavior) contribute to it simultaneously [14, 15]. The Job Demands–Resources (JD-R) model can be used to interpret the results: high demands (dementia-specific care burden, time pressure, emotional/psychological strain) and low resources (personal/material conditions, managerial and team support, role clarity, training) together increase the likelihood of exhaustion [9].

### Dementia-specific symptom burden

The 12-item symptom burden block showed strong internal consistency (Cronbach’s α = 0.949). Based on the descriptive results, several symptoms that are particularly burdensome in everyday care (e.g. aggression, apathy/depression, repetitive questioning, lack of inhibition, sleep-wake disturbances) appeared with high average burden values. These patterns are consistent with the international literature, which states that behavioral and psychological symptoms of dementia (BPSD) are extremely common during the course of the disease and significantly increase both care complexity and caregiver distress [16]. A practical implication is that BPSD-focused competency development may represent an important element within broader multi-level strategies aimed at reducing caregiver burden [6]. Moreover, within a Job Demands–Resources perspective, staff strain is shaped by the interplay of high demands and limited organizational resources (e.g., time pressure, emotional load, inadequate staffing, low managerial and team support, unclear roles, and restricted opportunities for training and recovery). This interplay can amplify exhaustion and undermine sustained improvements in care [9].

### Workload and frequency: the dominance of “demands” and the role of rest

In the ordinal regression model, higher perceived stress and more frequent care difficulties were associated with higher odds of being in a more severe burnout category, which is consistent with the JD–R health-impairment process linking sustained job demands to exhaustion and burnout development [9]. However, given the cross-sectional design, these findings should be interpreted as associations rather than directional effects. The non-significant correlation of the variable indicating the rarity of rest can be interpreted in several ways: (i) it is possible that the measure of “rest” did not capture the quality of psychological detachment; (ii) based on the “recovery paradox”, those who need it the most have the most difficulty regenerating; (iii) the effect of rest may be exerted indirectly, through intermediate mechanisms. When interpreting the results, it should be taken into account that recovery from work-related strain is multidimensional, and different recovery processes and experiences should be distinguished (e.g., psychological detachment, relaxation, mastery/control, and broader resource replenishment) [17–19]. In addition, contemporary work-recovery research highlights sleep – alongside psychological detachment – as a key recovery process, particularly under sustained stressor exposure [18, 20]. According to the “recovery paradox”, workers facing persistently high workloads are often those who have the least opportunity to recover effectively; therefore, self-reported “frequency of rest” may be an imperfect proxy for actual recovery, and its association with exhaustion may be complex (and potentially non-linear) [20]. Consequently, future models may benefit from operationalizing recovery in a more multidimensional way (e.g., psychological detachment, sleep quality, and non-work resource replenishment), because mere “frequency” is not necessarily equivalent to effective recovery [18, 19].

### The protective role of burnout-related knowledge

Self-reported prior learning about burnout showed a very strong negative association with burnout severity (OR ≈ 0.06–0.08 across models). The magnitude of this association suggests a substantial difference between those reporting prior learning and those not reporting such learning, but causal interpretation is not possible based on this research. This correlation fits with the idea that training and psychoeducation act as a “resource”: it can improve early symptom recognition, increase coping repertoire, and reduce feelings of helplessness. The effectiveness of burnout-reducing interventions among nurses – particularly person-directed psychoeducational approaches (e.g., mindfulness-based and cognitive-behavioral interventions) – is supported by systematic reviews and meta-analyses [21, 22]. However, cross-sectional studies are not suitable for determining the direction of causality. It may also be that employees with lower burnout or a higher sense of control are more likely to participate in training. The “learning” item may also be due to differences in institutional culture and leadership. Therefore, this result can be considered a strong, but cautious, indication that warrants a prospective study.

### Experience and burnout

Several categories of experience (especially 11–20 years and 21–30 years) were associated with an increased risk of burnout compared to the reference category. This pattern may reflect cumulative exposure to emotionally demanding caregiving conditions [14]. According to international burnout literature, burnout in an organizational context can become entrenched when a sustained mismatch persists between job demands and available resources. This process is often accompanied by a reduced sense of efficacy (ineffectiveness), which is considered a core component of the burnout experience [23]. Based on this, the practical interpretation suggests that targeted retention and support packages (career paths, rotation, competency development, psychological support, management feedback) in the mid-career stage can be particularly profitable.

### Coping typology

According to the coping typology in the sample, the vast majority indicated active coping, while a smaller but professionally significant group indicated the impossibility of coping (“I don’t have the opportunity”; “I’m too busy”). The category of “no opportunity” in the logic of JD-R may indicate a lack of resources: not necessarily a lack of coping skills, but an insufficiency of structural conditions (time, work schedule, staff, managerial support) [9]. This is consistent with the view that staff stress in challenging situations is context-dependent and shaped by personal appraisal; when organizational job resources are constrained (e.g., limited time, staffing, and managerial support), staff may have fewer opportunities to apply coping strategies effectively, which can appear as a “coping access” problem [15, 9]. This group may represent a priority target for intervention, particularly if structural constraints limit the effective use of coping strategies.

### Organizational and quality of care consequences

Among the items of care difficulties, the lack of human resources, cooperation and competences were prominently mentioned. This reinforces the fact that preventing burnout is not only a question of individual resilience, but also an issue of institutional operation and quality assurance. According to international evidence, organisational-level person-centred care in dementia services – typically implemented through staff education/training and leadership-supported organisational change – can improve outcomes for people living with dementia (particularly quality of life) and may enhance staff person-centred care behaviour [24, 25]. These results are broadly consistent with the priorities outlined in the WHO Global Action Plan on the Public Health Response to Dementia 2017–2025 [26].

### Practical suggestions

Given that mixed (multi-component) psychoeducational approaches have shown advantages over single-component interventions in reducing nurse burnout, a component-based intervention package may be a pragmatic option [21, 22]:

1. **BPSD management competencies** (e.g., evidence-based non-pharmacological strategies, de-escalation, communication, environmental/routine adaptation) [7] – as BPSD are among the most complex and stressful aspects of dementia care and a major driver of caregiver strain.
2. **Strengthening workplace resources** (team communication, clarification of roles, management support, case discussion/supervision) [18] – because staff strain in long-term dementia care is consistently linked to organizational conditions such as perceived support/leadership and care-home environment [16].
3. **Improving the quality of regeneration** (psychological detachment, detachment-based techniques, structured provision of rest) [17, 18, 20] – because recovery is multidimensional and recovery processes should be distinguished from mere time off (e.g., detachment vs. other recovery experiences), while sleep is repeatedly highlighted as a core recovery process. In line with the “recovery paradox,” staff exposed to persistently high workloads may have the least opportunity to recover effectively; therefore, the association between self-reported “rest frequency” and exhaustion may be complex, and frequency alone is not necessarily a valid proxy for effective recovery [20].

### System-level (policy) outlook

In residential dementia care, burnout is not only an individual well-being outcome but also a workforce sustainability issue. This is because burnout has been shown to be associated with job withdrawal outcomes such as absenteeism, intention to leave, and turnover [23]. The WHO dementia framework also emphasizes support for caregivers and the development of service capacities [26]. In the Hungarian care system, this may be particularly relevant by coordinating training, organizational capacity, and PCC-oriented developments. Based on the results, monitoring institutional-level training coverage (with a focus on burnout and BPSD) and reducing the proportion of the “I have no way to cope” group can be presented as well-defined, measurable development goals.

### Limitations and strengths

Several limitations affect the interpretation of the presented results and conclusions. First, the cross-sectional survey does not allow for a clear determination of the causal direction (e.g. in the case of the relationship between “learning” and burnout). Second, the data collection was limited to one county and residential institution types, and the sampling was not considered random - generalizability is limited. Third, all variables were based on self-report. Fourth, the burnout outcome variable is a multiple-choice item with a severity indicator of 0–3, which represents a pragmatic operationalisation and does not replace a validated multidimensional instrument such as the Maslach Burnout Inventory (MBI) or the Oldenburg Burnout Inventory (OLBI). Although the Brant test indicated a statistical deviation from the proportional odds assumption in the single-level model, the consistency of effect directions and magnitudes across the multilevel model supports the substantive robustness of the findings. However, the strengths include the large number of items (almost complete coverage), the data collection covering several institutions, and the excellent reliability and well-structured dimensionality of the scales of caregiving difficulties and symptom burden. The consistency of the results with the results of the international literature provides a solid basis for the precise definition of intervention points and for a possible further longitudinal research. The latter means a measurement carried out at least at two points in time to test whether workload and the frequency of difficulties prevent the exacerbation of burnout in time, and whether training/learning really acts as a causal protective factor. A step forward could be to strengthen the measurement of burnout with a validated tool and to develop complex models that simultaneously address institutional characteristics (capacity, staffing ratio, training practices, leadership style) and individual factors, in line with the JD-R framework.

## Conclusion

This study investigated burnout severity among nurses and caregivers in residential dementia care within a Job Demands–Resources framework. Higher subjective workload, more frequent care-related difficulties, and perceived constraints in accessing coping opportunities were consistently associated with increased burnout severity, while prior learning about burnout showed a protective association. Multilevel modelling indicated that a substantial proportion of variance was attributable to between-institution differences, underscoring the importance of organisational context.

These findings suggest that burnout in dementia care is shaped by both individual-level demands and structural resource conditions. Preventive strategies should therefore address workload management, dementia-specific competencies, and organisational support mechanisms. Longitudinal research is needed to clarify causal pathways and evaluate the impact of institutional reforms on burnout severity.

## Supplementary Information

Below is the link to the electronic supplementary material.

Supplementary Material 1

## Data Availability

The data were collected through a custom-developed web interface, where caregivers provided information anonymously. Questionnaire responses are stored in a database located on a password-protected laptop. Access to this database is restricted exclusively to HK and PT. The resulting datasets are stored in a private database and remain accessible upon research request. These data have not been utilized for other research or any other external publications. For the purposes of analysis, the data were exported in Excel format. All participants received detailed written and verbal information regarding the study. They provided their written informed consent to the data management terms and conditions, which are maintained and stored by HK. The data collection process ensured full anonymity, and all information is handled in strict accordance with data protection regulations.

## Abbreviations

AIC: Akaike Information Criterion
ANOVA: Analysis of Variance
BPSD: Behavioral and Psychological Symptoms of Dementia
CI: Confidence Interval
CLMM: Cumulative Link Mixed Model
GVIF: Generalized Variance Inflation Factor
JD-R: Job Demands-Resources model
KMO: Kaiser–Meyer–Olkin measure
LTC: Long-Term Care
MBI: Maslach Burnout Inventory
OR: Odds Ratio
PCA: Principal Component Analysis
PCC: Person-Centered Care
POLR: Proportional Odds Ordinal Logistic Regression
WHO: World Health Organization
