# Supplementary material for: Burnout severity in residential dementia care: individual demands and institutional-level variation in a Hungarian cross-sectional study
Source: BMC Nurs. 2026 Mar 30;25:432. doi: 10.1186/s12912-026-04579-y (PMC13159206; doi:10.1186/s12912-026-04579-y)
Supplement: Supplementary file 1 — Supplementary Material 1 [file 12912_2026_4579_MOESM1_ESM.docx]

**Dear Participant,**

Nowadays, the number of people living with dementia is rising worldwide.

Due to their unusual intellectual, communication, emotional, and behavioural manifestations, they appear strange to people and communities, occasionally causing fear; thus, we cannot speak of social stigma for these patients.

Literature references and research prove that nursing and caring for people living with this type of illness presents many difficulties even for a portion of health and social care professionals.

In our study conducted by the Department of Nursing at the Faculty of Health Sciences, Semmelweis University, we wish to assess the knowledge and personal attitudes of healthcare and social care professionals regarding dementia-related diseases, the special needs of patients, and the professional cooperation between those providing care.

Our goal is to explore, based on the study, the knowledge and views of nursing and care professionals regarding the disease, the conditions and difficulties of care, as well as their personal attitudes, strategies, and potential prejudices useful for care.

By summarizing the obtained information, we intend to make proposals later for inclusion in the curriculum of vocational and continuing education for health and social care professionals.

We hope that incorporating novel knowledge, good and useful practices, and effective methods can reduce the reservation and prejudice of nursing and care professionals towards patients in a state of dementia, and that possessing a much more sensitive, accepting attitude will lead to greater sensitivity towards the people living with dementia. A nursing and care strategy built on proper foundations and a more accepting personal attitude provide, on the one hand, the opportunity for higher quality, more humane care, and on the other hand, significantly facilitate the work of those working in this professional field.

For this, we ask for your supportive assistance by filling out the questionnaire below.

Filling out the questionnaire is done anonymously and is not too time-consuming.

Thank you for helping to realize the goals of the research with your honest answers!

**The following instructions to assist with completion are as follows:**

1. Answering questions marked with an asterisk * is mandatory in all cases, and we ask that you provide an answer for every question.
2. Please mark the appropriate boxes with an x.
3. Answering certain questions is done on a scale ranging from 1 to 5, where 1 is the lowest and 5 is the highest rating.
4. For some questions, only one answer may be given; for others, multiple answers are possible; the request for multiple answers is indicated below the question.
5. For some questions, you must write in the answer; please write your answer legibly, possibly using block letters!

QUESTIONNAIRE

**Personal Data**

**1. Gender***

- Female
- Male

**2. Age***

- 21 - 30
- 31 - 40
- 41 – 50
- 51 – 60
- 61-70

**3.Education level (mark all qualifications)***

- Social Caregiver and Nurse (Primary School based)
- Social Caregiver and Nurse (Secondary School based)
- Healthcare Vocational School
- Healthcare Vocational School with Diploma
- College (BSc), major: ………………………………….
- University (MSc), major: ………………………………….
- Specialized Dementia Care Qualification
- Other health or social qualification(s) …………………………………………………………..

**4. How long have you been working in the social field as a nurse or carer?***

- Under 1 year
- 1 – 5 years
- 6 – 10 years
- 11 – 20 years
- 21 – 30 years
- 31 – 40 years
- Over 40 year

**5.Please indicate where the settlement where the institution is located can be classified among the following answer options!***

- City/Town
- Commune/Municipality
- Village

**6.Please indicate how many beds (capacity) the institution where you work has!***

- 1 - 25 beds
- 26 – 50 beds
- 51 – 100 beds
- Over 100 beds

**7.Have you ever met a person living with dementia in your life?*** (Multiple answers possible)

- Yes, in my family
- Yes, in my neighbourhood
- Yes, in my circle of friends
- Yes, during my work
- In wider social settings e.g., club, Alzheimer café
- I have not yet met a person living with dementia

Other:…………………………………………………………………………………

**8. Please briefly write down the three adjectives or thoughts that first come to your mind regarding a person living with dementia!**

………………………………………………………………………………………………………………………………………………………………………………………………………………………………………………………………………………………………………

**9. Please rate on a scale of 1 to 5 to what extent the factors listed below cause you difficulty during the nursing and care of dementia patients! (**The marking is done on a scale ranging from 1 to 5, where 1 is the lowest and 5 is the highest rating.)

| **Factor complicating nursing and care** | **Marking on the 1-5 scale** |
| --- | --- |
| Lack of up-to-date theoretical knowledge about the disease and its specific care | 1…2…3…4…5 |
| Inadequacy of environmental conditions necessary for special care | 1…2…3…4…5 |
| Insufficient material resources necessary for special care | 1…2…3…4…5 |
| Inadequate staffing levels, low number of care providers | 1…2…3…4…5 |
| Practical professional knowledge of care staff is not sufficient | 1…2…3…4…5 |
| No proper cooperation among colleagues | 1…2…3…4…5 |
| Caring of bedridden patients incapable of self-care represents a significant physical burden | 1…2…3…4…5 |
| Special care needs of hard-to-manage, confused patients require continuous readiness and attention | 1…2…3…4…5 |
| Sleep-wake cycle disturbances frequently occurring in patients disrupt the work schedule | 1…2…3…4…5 |
| Occupation and daily routine of patients are not sufficient | 1…2…3…4…5 |
| Lack of cooperation from relatives, or rejective behaviour stemming from lack of knowledge | 1…2…3…4…5 |
| I have become physically exhausted by this work, I can no longer bear this strain | 1…2…3…4…5 |
| I am mentally tired of this work, I am burnt out | 1…2…3…4…5 |
| Other………………………………………………………………… | 1…2…3…4…5 |

**10. Please rate the symptoms listed below based on how much the symptom makes patient care difficult during nursing and care!** The marking is done on a scale ranging from 1 to 5, where 1 is the lowest and 5 is the highest grade.

| **Name of symptom complicating nursing and care** | **Marking on the 1-5 scale** |
| --- | --- |
| Neglect of personal hygiene | 1…2…3…4…5 |
| Physical deterioration, immobility | 1…2…3…4…5 |
| Repetitive questioning | 1…2…3…4…5 |
| Verbal aggression/shouting | 1…2…3…4…5 |
| Physical aggression | 1…2…3…4…5 |
| Resistant behaviour/lack of cooperation | 1…2…3…4…5 |
| Wandering | 1…2…3…4…5 |
| Sleep disorders | 1…2…3…4…5 |
| Confusion | 1…2…3…4…5 |
| Depression, apathy | 1…2…3…4…5 |
| Hallucinations, delusions | 1…2…3…4…5 |
| Lack of inhibition, sexual behavioural disorders | 1…2…3…4…5 |
| Other symptom: ……………………………………………. | 1…2…3…4…5 |

**11. Please rate on a scale of 1 to 5 what type and degree of burden nursing and caring for dementia patients means to you.** The marking is done on a scale ranging from 1 to 5, where 1 is the lowest and 5 is the highest grade.

| **Burden related to nursing and care** | **Marking on the 1-5 scale** |
| --- | --- |
| ο Physical | 1…2…3…4…5 |
| ο Cognitive | 1…2…3…4…5 |
| ο Psychological | 1…2…3…4…5 |

**12. How often do you experience difficulties while caring for patients with dementia?**

- Rarely
- Every day
- One or two times a week
- I do not feel difficulty

**13. Do you have the opportunity to rest after work?**

- Yes, every day
- One or two times a week
- A few times a month
- I have no opportunity to rest at all

**14. I feel that during my previous studies I have already acquired the appropriate theoretical and practical knowledge regarding the treatment of and communication with patients living with dementia.*** (Please rate the following statement on a scale of 1 to 5, where 1 is the lowest and 5 is the highest rating, circle the appropriate number!)

1 2 3 4 5

**15. If I understood the illness of persons living with dementia better, I would nurse the people living with this condition with greater compassion. *(**Please rate the following statement on a scale of 1 to 5, where 1 is the lowest and 5 is the highest rating, circle the appropriate number!)

1 2 3 4 5

**16. In your opinion, do you possess sufficient professional knowledge/information about dementia?** (Multiple answers may be marked.

- I possess this knowledge
- I possess knowledge, but I feel it is insufficient
- I possess knowledge, but I feel it is outdated
- I do not possess sufficient knowledge

**17. Would you need to acquire new knowledge, professional development regarding modern nursing-care methods for dementia patients?**

- Yes
- No
- Other …………………………………………………………………………………….

**18. Have you attended further training on the subject of dementia?** (Multiple answers may be marked)

- Yes, it was useful, I learned a lot, modern, fresh information was presented, I can use it in my work.
- Yes, what I experienced there helped in attuning to the patients' needs, I became more accepting towards them and towards the relatives.
- Yes, but I did not feel it was useful, the knowledge did not provide any new information.
- Yes, but I cannot apply the information presented in my daily practical work.
- I have not been, no relevant training was available for me
- I have not been it was not possible to leave work
- I have not been, the training was held at an inaccessible location
- Other: …………………………………………………………………………………..

**19. Do you find the staff number at your workplace sufficient for the care of dementia patients?** (Please rate on a scale of 1 to 5!)

1 2 3 4 5

**20. If you could, what environmental conditions would you change for the sake of quality patient care?**

- Primary: ………………………………………………………………………………
- Secondary: ……………………………………………………………………………
- Tertiary: ………………………………………………………………………………
- Other comments: ……………………………………………………………………….

**21. If you could, what material conditions would you change for the sake of quality patient care?**

- Primary: ………………………………………………………………………………
- Secondary: ……………………………………………………………………………
- Tertiary: ………………………………………………………………………………
- Other comments: ……………………………………………………………………….

**22. What kind of qualified professional(s) would be needed at your workplace for higher level care?**

- Primary:………………………………………………………………………………
- Secondary:……………………………………………………………………………
- Tertiary:………………………………………………………………………………
- Other comments:………………………………………………………………………

**23. How do you judge the mental hygiene care and meaningful activities of dementia patients?** (Multiple answers may be marked)

- The programs are varied
- Consistency is ensured through a well-established daily routine
- There is little occupation/activity
- Activities are provided, but they are not appropriate for the patients’ needs

**24. List which personal traits (attitudes) are necessary to some extent for the care of dementia patients!** (Fill in the table, the scale ranges from 1 to 5, where 1 means the extent is not necessary at all, and 5 is the most important. Mark the appropriate values!)

| **"Abilities, skills" related to work** | **Scale: 0-1-2-3-4-5** | **"Abilities, skills" related to work** | **Scale: 0-1-2-3-4-5** |
| --- | --- | --- | --- |
| Adaptability | 0-1-2-3-4-5 | Monotony tolerance | 0-1-2-3-4-5 |
| Friendliness | 0-1-2-3-4-5 | Persistence in work | 0-1-2-3-4-5 |
| Speech comprehension | 0-1-2-3-4-5 | Independent problem solving | 0-1-2-3-4-5 |
| Empathy | 0-1-2-3-4-5 | Self-knowledge | 0-1-2-3-4-5 |
| Cooperation skills | 0-1-2-3-4-5 | Self-regulation | 0-1-2-3-4-5 |
| Emotional resilience | 0-1-2-3-4-5 | Self-control | 0-1-2-3-4-5 |
| Emotional balance | 0-1-2-3-4-5 | Precision, self-check | 0-1-2-3-4-5 |
| Sense of responsibility | 0-1-2-3-4-5 | Problem-solving ability | 0-1-2-3-4-5 |
| Physical stamina | 0-1-2-3-4-5 | Realistic situation recognition ability | 0-1-2-3-4-5 |
| Humanity | 0-1-2-3-4-5 | Orderliness | 0-1-2-3-4-5 |
| Manageability / Receptiveness to direction | 0-1-2-3-4-5 | Personality stability | 0-1-2-3-4-5 |
| Judgment | 0-1-2-3-4-5 | Organizational skills | 0-1-2-3-4-5 |
| Networking skills | 0-1-2-3-4-5 | Resourcefulness | 0-1-2-3-4-5 |
| Manual dexterity | 0-1-2-3-4-5 | Sustained concentration (attention) | 0-1-2-3-4-5 |
| Communication skills (verbal and "body language") | 0-1-2-3-4-5 | Professional distancing | 0-1-2-3-4-5 |
| Consistency | 0-1-2-3-4-5 | Stress tolerance | 0-1-2-3-4-5 |
| Sense of community | 0-1-2-3-4-5 | Cleanliness | 0-1-2-3-4-5 |
| Creativity | 0-1-2-3-4-5 | Confidentiality | 0-1-2-3-4-5 |
| Resilience to failure | 0-1-2-3-4-5 | Evident-based approach | 0-1-2-3-4-5 |
| Logical thinking | 0-1-2-3-4-5 | Patience | 0-1-2-3-4-5 |
| Reliability | 0-1-2-3-4-5 | Innovativeness | 0-1-2-3-4-5 |
| Observational skills | 0-1-2-3-4-5 | Leadership skills | 0-1-2-3-4-5 |
| Other | 0-1-2-3-4-5 | Other | 0-1-2-3-4-5 |

**25. How accepting are you with dementia patients?** (Please rate on a scale of 1 to 5!

1 2 3 4 5

**26. Do you know communication, distraction, tension-relieving strategies that work well regarding patient care?** (Multiple answers may be marked)

- Yes, I am familiar with them from professional literature
- Yes, I know them from the continuing education programs
- I know them and can appropriately use them during my nursing, care work
- Unfortunately, I do not know them sufficiently
- Other …………………………………………………………………………………

**27. What kind of relationship are you generally able to establish/maintain with the patients' relatives?** (Multiple answers may be marked, write in the chosen places for how many patients the statement is true!)

| **Statements** | **Number of patients’ relatives** |
| --- | --- |
| We are able to cooperate for the sake of the patient, to communicate appropriately | …….. people |
| The relationship and communication between us is good, but their expectations are often excessive and unrealistic | …….. people |
| Cooperation is difficult, their expectations are unrealistic, multiple times excessive, overly demanding | …….. people |
| There is barely any relationship, the relatives only seemingly care about the patient | …….. people |
| There is no relationship, nobody visits the patient | …….. people |
| Other statements: ………………………………………………. |  |

**28. Do you have the opportunity to discuss, to ventilate problems related to your work?** (Multiple answers may be marked.)

- Yes, at staff meetings or shift handovers
- Yes, among colleagues
- At home with my family, friends
- There is no opportunity

**29. Increased load can cause burnout, do you know the symptoms of the condition?** (Multiple answers may be marked.)

- Yes, I learned, read about it
- Yes, I am starting to notice these symptoms on some of my colleagues too
- Yes, unfortunately I am starting to notice these symptoms on myself too
- Yes, and since I am starting to notice these symptoms on myself too, I am thinking about changing careers
- None of the above

**30. What methods do you use so that you are physically and mentally able to perform this difficult work without health damage?** (Mark, or write your answers in the dotted places! Multiple answers are possible.)

- Sports, physical recreation (specify type of activity)…………………………..
- Regular hobby or leisure activity (exactly what) …………………………………
- Prioritizing family life, I “do not take the work home”
- Extensive circle of friends, social programs
- Community involvement or club activity (exactly what) …………………………………
- Professional help, using the help of a psychologist, psychiatrist, supervision
- I have no opportunity, I am overwhelmed with work
- I do not think I need any such activity
- Other comments: …………………………………………………………………

Thank you very much for your cooperation and for participating in my research!
